# The ability to benefit from an intervention to encourage use of treadmill workstations: Experiences of office workers with overweight or obesity

**DOI:** 10.1371/journal.pone.0228194

**Published:** 2020-01-28

**Authors:** Frida Bergman, Kerstin Edin, Rebecka Renklint, Tommy Olsson, Ann Sörlin

**Affiliations:** 1 Department of Public Health and Clinical Medicine, Umeå University, Umeå, Sweden; 2 Department of Nursing, Umeå University, Umeå, Sweden; 3 Department of Community Medicine and Rehabilitation, Umeå University, Umeå, Sweden; University Lyon 1 Faculty of Dental Medicine, FRANCE

## Abstract

One way to increase physical activity in offices is to install treadmill workstations, where office workers can walk on a treadmill while performing their normal tasks. However, the experiences of people using these treadmill workstations over a long period of time is not known. In this 13-month study, we explored the experiences of office workers with treadmill workstations available in their offices. After completing a larger randomized controlled trial with 80 office workers ages 40 to 67 years with overweight or obesity, we interviewed 20 participants from the intervention group, using a semi-structured interview guide. Data were analyzed using a grounded theory approach with constant comparison of emerging codes, subcategories, and categories, followed by connecting the categories to create a core category. The core category is described as the “Ability to benefit.” Although all participants had a rather high motivational level and pre-existing knowledge about the health benefits of increasing physical activity at work, they had different capacities for benefiting from the intervention. The categories are described as ideal types: the *Convinced*, the *Competitive*, the *Responsible*, and the *Vacillating*. These ideal types do not represent any single participant but suggest generalized abstractions of experiences and strategies emerging from the coding of the interviews. One participant could easily have more than one ideal type. Because of differences in ideal type strategies and paths used throughout the course of the study, participants had different abilities to benefit from the intervention. Knowledge regarding the ideal types may be applied to facilitate the use of the treadmill workstations. Because different ideal types might require different prompts for behavior change, tailored intervention strategies directed towards specific ideal types could be necessary.

## Introduction

Modern society fosters sedentary behavior in many domains, especially in the workplace, where the number of sedentary jobs has steadily increased over the past decades, enhanced by an increase in computerized work [1]. Sedentary behavior is defined as activities with a low energy expenditure (≤1.5 metabolic equivalents) while in a seated, lying or reclining posture [2]. Sedentary behavior increases risk for several adverse health outcomes, such as the development of type 2 diabetes and all-cause mortality [3, 4]. Thus, it is important to find strategies that can decrease time spent in sedentary behavior and promote a more active way of performing regular office work.

Treadmill workstations consist of a treadmill desk that office workers use while at their computer. We have previously reported the study setup and results from the Increasing Physical Activity (Inphact) Treadmill Study. For this randomized controlled trial, we installed treadmill workstations in offices and compared use of this setup for 13 months to regular office work [5, 6]. We have shown that it is indeed possible to increase time spent in physical activity in offices with such workstations [6]. Facilitating their implementation, however, requires further exploration of factors that influence their use.

At short-term follow-ups, participants reported feeling positive and enthusiastic about treadmill workstations, saying that they would continue to use them if they were available [7, 8]. The reactions and experiences of individuals using treadmill workstations over a longer period of time are not as clear. In one small study, investigators evaluated facilitators and barriers in four predefined areas, namely usability, safety, comfort, and productivity, for five female office workers who used treadmill workstations for 6 months. The results suggested that common barriers were related to office desk setup and the effect of the walking noise on communication. Facilitators included the feeling of enjoyment and more energy after using the treadmill for a period of time [9]. In another study in a health insurance company, researchers investigated adherence issues during implementation of shared treadmill workstations for 6 months among office workers with overweight or obesity. Common reasons for not using the treadmill workstation were work conflicts, being out of the office, and illnesses/injuries [10]. The shared treadmill workstations were to be used by many employees, and scheduling was a noted difficulty when participant work schedules had to be considered [10]. Individual treadmill workstations might involve other difficulties, but what these are requires further investigation.

In this qualitative study, we sought to add to the knowledge base and explore the factors that influence decisions for those who have access to an individual treadmill workstation for an extended period of time. Our objective was to explore the experiences of office workers who during 13 months had access to treadmill workstations, which were installed in their offices.

## Methods

### Setting

This qualitative interview investigation was part of the Inphact Treadmill Study, performed in Umeå, Sweden, during 2014–2015 [5]. Umeå is a small university city in northern Sweden and at the time of this study had a population of 118,000 inhabitants. The labor market is diverse and has a large number of office workplaces.

### The Inphact Treadmill Study

In the Inphact Treadmill Study, 80 participants were individually randomized to an intervention or a control group. Members of the research team contacted different workplaces in Umeå with information about the study. If company management showed interest, a meeting was held with the employees to familiarize them about the health risks of sedentary behavior along with providing information about the study. Employees who were interested in participating were screened via a telephone call and a visit to a hospital, where blood samples were taken. They also completed questionnaires regarding, e.g., anxiety, depression, and stress-related exhaustion disorder [5].

Healthy office workers without severe disease and whose work tasks were most sedentary were included. In addition, for inclusion, their body mass index had to be from 25 to 40 kg/m^2^, classified as overweight or obesity, and their age had to be from 40 to 67 years. Half of the group started the study in April 2014 and the other half started in October 2014.

After the baseline measurements, participants were randomized to the intervention or the control group. All participants received a health consultation with a trained registered nurse after randomization. In this consultation, they discussed some of their baseline measurements and received general recommendations about physical activity and diet. Those randomized to the control group continued to work at their sit–stand table as usual, whereas those randomized to the intervention group had an individual treadmill workstation to use for 13 months. The workstation consisted of a treadmill adapted for use at a sit–stand office desk and was only made for walking, not jogging. The participants were asked to use this treadmill for at least one hour per day at a time of their choice. The treadmill could be moved when not in use.

Before starting to use the treadmill workstation, the participants received help with installation from an experienced physiotherapist. Technical support was available for any problems that occurred with the treadmill. The participants in the intervention group also received supportive emails on four occasions during the study period. These emails provided information about the health benefits of reducing sedentary behavior. Different examinations were performed during working hours at baseline and at the 2-, 6-, 10-, and 13-month follow-ups. The examinations included physical activity and sedentary behavior measurements; general body examinations (including anthropometric measurements); body composition and blood samples; cognitive function tests; and structural and functional magnetic resonance imaging. Participants also completed a food diary and questionnaires concerning, among other things, experienced pain and stress. These measurements are described in more detail in a previous publication [5].

### Interview data collection

For this interview study, we gathered the data through 20 individual in-depth interviews with participants from the intervention group (11 men and 9 women). The interviews were conducted in the spring and autumn of 2015, immediately after the Inphact Treadmill Study was finished [5]. Participants in the first intervention group ended the study in May or June, and all of these participants were the first to be invited to participate in the interviews. A few participants from the second group, who ended the study in November or December, were also asked to participate, based on convenience sampling. A total of five participants declined to participate in the interviews; one from the first group and four from the second group. For these interviews, we used a semi-structured interview guide (S1 Table) drawn from earlier research [9] with the aim of gaining deeper insight into the respondents’ experiences of the treadmill workstation. Four female interviewers, all from the field of physiotherapy, conducted these interviews: one researcher with great experience in conducting interviews, while the other three were students (one was a PhD-student) with limited experience of conducting interviews. The students were trained before and also supervised during the interviews. The participants could choose the location of the interview from the Clinical Research Center at Norrlands University Hospital, the Northern Behavioral Center at Umeå University, or their workplace. The majority of the interviews took place at the workplaces. The interviews were between 13 to 34 minutes long and were first recorded and later transcribed verbatim.

### Data analysis

We analyzed data from the interviews using a modified form of grounded theory, inspired by Charmaz [11]. The modification in this case consisted of omission of comparisons and analysis of data between each individual interview; thus, by definition within grounded theory, an emergent design could not be used. We made this choice for practical reasons because we wanted to interview participants directly after their treadmill walking experience. The researcher and PhD-student from the field of physiotherapy, who had conducted the interviews, one researcher from the field of nursing with great experience of qualitative research and one medical student later performed a triangulation process with thorough processing and comparison of data. They coded the interviews individually for later coding comparison, and together searched for emerging patterns in the coding until reaching agreement. The researcher who was involved as interviewer carried out the complete and final part of the selective coding into subcategories, categories, and a final core category. To further describe the substance of the categories during the coding process, properties of the categories were identified together with their different dimensions, describing how the properties may vary on a continuum [11]. All co-authors involved in the triangulation process agreed to these final interpretations done in the final part of the coding. The interviews and all of the coding were performed in Swedish, and a professional translator translated all the quotes into English. When quoted here, the participants appear with fictitious names.

### Ethical considerations

The research protocol for the Inphact Treadmill Study was approved by the Regional Ethical Review Board in Umeå. All participants gave oral and written informed consent to participate. The managers of the participating companies had no access to any of the data collected in the study. Data were handled with confidentiality according to ethical guidelines [12]. Participation in the study was voluntary, and all participants were informed that they could withdraw from the study at any time, without providing any reason for withdrawal.

## Results

Baseline characteristics of the interviewed participants are given in Table 1.

**Table 1:** Demographic data at baseline for the interviewed participants.

**Age [years]**, mean (distribution)	53.2 (40–62)
**Males/females**, n (%)	11/9 (55/45)
**BMI [kg/m**^**2**^**]**, mean (distribution)	29.8 (25.5–38.8)
**Education level**, n (%)	
Compulsory	0 (0)
Upper secondary	8 (40)
University	7 (35)
Other tertiary	5 (25)
**Physical exercise**, n (%)	
Never	6 (30)
Not regularly	3 (15)
1 time/week	2 (10)
2–3 times/week	7 (35)
>3 times/week	2 (10)
**Self-reported health**, n (%)	
Excellent	0 (0)
Very good	10 (50)
Good	7 (35)
Fair	2 (10)
Bad	1 (5)

In the following section, we describe the core category, the four identified categories presented as ideal types, and the properties and dimensions of the categories. Table 2 shows examples of the coding and the subcategories and how they formed the categories and core category.

**Table 2:** Examples of the codes and subcategories, and the final categories/ideal types and core category.

Codes	Subcategories	Categories–Ideal types	Core category
High activity	Active daily	*The Convinced*	Ability to benefit
Prior standing behavior			
Contribute to health	Strong belief in the cause		
Activity needed			
Activity on paid working hours	Use the opportunity		
Better health			
Show the amount	Comparisons	*The Competitive*	
Higher speed than the others			
All tasks can be completed	Solution focused		
No one is disturbed			
Register time	Record-keeping		
Own file			
Health is important	Participate for the benefit of research	*The Responsible*	
Contribute to science			
Had promised	Duty		
Wants to complete			
Pressure to perform	Shame		
Lazy			
A lot of support	Hopeful	*The Vacillating*	
Help			
Need more pep talk	Reluctant		
Fed up			
Pain	Disappointed		
Dual tasking difficulty			

### The core category

The core category is labeled the “Ability to Benefit.” All participants were volunteers who were positive about the idea that increased physical activity during work hours could support better health. Their motivational level in the beginning of the study was high because they chose to participate in this rather intensive study. However, not all participants adhered and responded in the same way to the intervention. For this reason, they did not all have the same ability to benefit, despite being positive and having high initial motivation. This difference in ability to benefit from the intervention arose from differences in characteristics among the participants. The categories in this paper are presented below as ideal types, capturing these different characteristics within and between participants.

### Categories—Ideal types

Max Weber proposed the concept of ideal types, with a focus on social action [13] as was the object of our study. The ideal types in this study should be understood as generalized abstractions and strategies visualizing important properties that can be related to various behavioral models and motivational theories. The ideal types derived from the coding process of all interviews; they do not represent any particular participant. One person might very well have used strategies corresponding to more than one ideal type during the 13-month study period. The ideal types that emerged from our data were the *Convinced*, the *Competitive*, the *Responsible*, and the *Vacillating*. These different ideal types implicated different strategies and paths throughout the study, leading to variations in participants’ ability to benefit from the intervention, despite a universally high initial motivational level.

### The Convinced

This ideal type arose from notions about already living according to “best practice.” The activity level of the *Convinced* is already high, and there is a true belief and conviction that physical activity is an important habit for good health. This ideal type still wants to take all opportunities to improve. They are truly committed and need only confirmation to keep up the good work. They rarely encounter obstacles along the way and find the obstacles that do arise easy to conquer.

*No, but I think … In the first place, it was kind of like I thought it sounded interesting from the very beginning. And then I also saw the part that says, yeah, that you will get a checkup so that you know you are super healthy (laughs)*.

*But that subsided, and then there weren’t any problems at all; and when it started, I didn’t feel like that were any real problems, either. But then, I kind of run a lot. Well, a lot or a lot, but maybe a lot for my age. Or, I don’t know. But I do it about 3 times … or did it about 3 times a week*.

PER (male)

The motivational level of the *Convinced* can be pictured as exemplary. The behavior does not always change that much because their daily activity levels are already relatively high. This ideal type may be disappointed if their measured health data show no visible change.

### The Competitive

This ideal type loves the challenge. The *Competitive* keeps records of everything they do and is more interested in the path than in the result. This ideal type is always looking for a challenge and is generally willing to participate in any challenge that is presented. The personal goal might not be better health, but rather to beat the others–to walk longer, faster, or better. The *Competitive* likes to discuss their results with others, keep records and share ideas about how to overcome problems that arise.

*Yeah, but it was partly the novelty of it and partly my competitive spirit. That you want to achieve this … One person at our job created an Excel file where you could kind of chart a curve showing how far you had gone in relation to how far you should go and even the time and distance. So, it kind of spiced things up*.

BO (male)

*But then, you get kind of strange, weird. I can’t stop after 96 minutes. Let it go to 100 even. Yeah, but it is kind of like that. Even though it’s 4:30 pm and you promised your wife to pick her up at a certain time. So, you get there 5 minutes late … Then I can’t say that I just decided … I kept going 5 more minutes*.

PER (male)

The *Competitive* is aiming for the best ever result, striving uphill. Some of this type may be used to daily physical activity and others not as much. The change for the better, being faster, or walking longer is what is important.

### The Responsible

This ideal type knows what is best and is extremely interested in doing the right thing. They remain acutely aware of the promise they made to participate in the study and anxious that the researchers do not perceive them as a fraud. The *Responsible* often comments on having a feeling of shame if they miss the required hour on the treadmill workstation.

*Yeah*, *in September I promised a year*, *I think*, *so that … So*, *I have done an hour a day*. *Of course*, *not every day*. *Sometimes*, *I haven’t been as motivated*, *sometimes I was away or at meetings so that you almost can’t fit it in*. *But for the most part*, *I have tried*. *An hour*.

LINDA (female)

It has been a problem. And then I have had significantly more work travel than I expected when I enrolled in this study. So, sometimes it felt like a bit of an obligation, and I’ve felt somewhat bad that I didn’t do it some weeks because I was away for a week or so. And it doesn’t count, if you take an evening walk. It doesn’t compensate for it …

KICKI (female)

The *Responsible* can start at different levels and have various difficulties in reaching the goal of walking on the treadmill every day. But this ideal type always includes feelings of a duty to walk, not only for themselves but also for the research project. The *Responsible* has entered the program and needs to see their path through to the end.

### The Vacillating

This ideal type knows that something must be done to improve their health. Being highly committed, the *Vacillating* has high hopes for participation. As motivation drops and everyday life makes it more difficult to perform as intended, the reluctance to recognize their own behavior as the major cause of non-performance grows. In the end, this ideal type is mainly disappointed with the given conditions.

*It was … we were offered something and I matched the required criteria … And I thought this could be something positive for me because I have problems with my knees and am supposed to move around a lot*. *Walk and such*. *So*, *I thought this could be something that would be really good for me…*. *No*, *because I have these problems*, *some days I have a lot of trouble*, *and I think I move around a lot even so*. *So*, *I … I noticed that a lot of people here … And I have heard from the others who are doing it that it made them feel good*. *And I probably would have done it too*, *if I had been able to walk on it*.

DAG (male)

The path is never straight for the *Vacillating*. Their awareness is high, and their will is strong, but their ability to constantly run into various obstacles is worth noting. Many of the events that the other ideal types treat as facilitators, the *Vacillating* perceives as barriers.

### Properties and dimensions

The grounded theory analysis allowed us to identify process properties, giving further substance to the categories, which links to the different ideal types. The properties, together with their respective dimensions, are presented in Table 3. These properties are degrees of trust, willingness to challenge, sense of duty, and quality of awareness, all of which affect the development of strategies in the ideal types, which do not represent any single participant. Instead, one person might very well over time use different strategies corresponding to different ideal types.

**Table 3:** Properties and dimensions in each category/ideal type.

Category/Ideal types	Properties	Dimensions
The Convinced	Trust	Cognizance
		Beliefs
The Competitive	Challenge	Win
		Lose
The Responsible	Duty	Pride
		Shame
The Vacillating	Awareness	Hope
		Despair

## Discussion

We have explored the experiences of office workers who had 13 months of access to treadmill workstations, which were installed in their office. We characterize a core category, described here as the “Ability to Benefit.” The categories of ideal types we identified were the *Convinced*, the *Competitive*, the *Responsible*, and the *Vacillating*. These ideal types can be seen as generalized abstractions of experiences and strategies corresponding to the categories that emerged from the coding process of all interviews. They do not represent any particular participant. Even though all participants knew that increasing physical activity at work would benefit their health, different ideal types used different strategies and paths throughout the study, resulting in different abilities to actually benefit from the intervention.

According to the “Fogg Behavior Model” [14, 15], human behavior is a function of three factors: motivation, ability, and prompts. For behavioral change to occur, these three factors need to be present at the same time. Hence, even if the motivational level is high, the individual’s ability to perform the task still needs to be sufficient and vice versa–otherwise, the behavior will not occur. However, according to this model, behavioral change also requires a prompt at the right moment. Different types of prompts exist and should be used based on the person’s motivational and ability levels. For example, a person with high motivation and high ability might need only a “signal prompt,” i.e., a reminder, to perform the behavior. A person with high motivation but low ability might need a “facilitator prompt,” i.e., a prompt that makes the behavior in question easier, whereas a person with high ability but low motivation might need a “spark prompt”, including a motivational aspect [14, 15]. In the same way, different ideal types observed in our study might need different types of prompts to reach the goal of increasing physical activity at work. The properties, with their different dimensions, might be useful when aiming to find the most suitable prompts for physical activity among the different ideal types. For example, as the *Competitive* is driven by challenges, competitions might be the best way to trigger their behavior and stimulate their motivation. The *Vacillating* on the other hand might need prompts that increase both their awareness and motivation, such as support from colleagues, more frequent follow-ups, more pep talk or more training, whereas the *Convinced* might only need a confirmation every now and then to keep up the good work. For the *Responsible*, it might be important to see that the good work they are doing is being noticed by the management or team leader. Adherence to our and similar interventions might be improved by identifying different ideal type strategies among participants and working with prompts specific for these types to increase physical activity levels in offices. This needs to be tested in future studies.

Regarding ideal types that emerged from our data, they should not be seen as static, but rather as having the potential to change within and between individuals throughout life. In our results, the participant “Per” is for example cited in the quotes for both the *Convinced* and the *Competitive*, supporting this notion. Thus, although a factor might be considered a facilitator at one time point, it might be considered a barrier at another. For this reason, it is important to try to capture the different prompts that influence these ideal types and to adjust the intervention. This concept could be tested in future studies, e.g., by using an adaptive intervention design. In such a design, in order to improve efficacy, the intervention is adapted during the study period based on the response of participants. Participants not responding to a first intervention might, e.g., be randomized to another treatment option. Although more research is needed on this type of study design, preliminary research on weight management and sedentary behavior using an adaptive intervention design shows promising results [16] and could be tested together with our theory of ideal types.

The properties and dimensions of the ideal types that we found in our analysis–trust, challenge, duty, and awareness–work on different levels, including individual, social, organizational, and environmental levels. This pattern confirms the assumption that complex interventions such as ours can be difficult to interpret. Ecological models state that multiple factors on different levels affect human behavior, including at the individual, social, organizational, environmental, and policy levels. The social framework and physical environment of the different settings influence the amount of sitting that takes place [17]. According to this model, individual factors should not only be addressed separately but also combined with targeting physical and social environmental factors. Indeed, multi-component interventions produce greater reductions in sitting time compared to environmental or educational/behavioral interventions alone [18]. Such an approach should therefore be undertaken in future interventions.

Although the ideal types that we delineated are likely to be found within a well-motivated group of volunteers, obviously even this rather homogeneous group had different levels of motivation and strategies. The broader workforce likely represents a more heterogeneous sample of individuals with different levels of motivation, pre-existing knowledge, and interest in similar interventions. The results may differ if an ordinary office group would have access to treadmill workstations, without taking part in a research intervention, and the transferability to the general population and to companies not involved in such a study is thus unclear. Of note, research itself always influences the outcome. For example, one common characteristic of “the *Responsible*” was not wanting to disappoint the researchers, motivating this type to use the treadmill workstation because “they had promised to do so.” This effect adds to the challenge of implementing such interventions among people who are not participating in a research project. Brakenridge et al. [19] concluded that when the intervention is implemented from within the organization, such as being delivered by the head of the workplace wellness office, it is implemented in a way that suits the organization in question. Choosing this approach to implementation can make these interventions more easily scalable to other workplaces [19]. In our case, the researchers came from outside the organizations, performing the intervention with the installment of the treadmill workstations. If interventions like ours were introduced from within the participating organizations, the behavior change might be more sustainable. This approach also might make it easier to adapt the interventions toward the different ideal types, with prompts that target their needs.

### Limitations and strengths

This study is, to the best of our knowledge, the first to investigate the experiences of office workers with long-term access to a treadmill workstation, analyzed by a grounded theory approach. This approach gave us the opportunity to develop a theoretical model with the ideal types, which can be tested in future studies. A limitation of using ideal types is that doing so might simplify and polarize the studied phenomenon, while largely not taking interactions among types into consideration. The method is, however, a way to easily describe strategies and characteristics among the participants, assisting in understanding their behavior.

A qualitative study aims to explore and understand informant experiences in depth. In grounded theory, models are constructed using an abductive method, i.e., generating theory that is rooted in the data and then continuously testing this theory with new informants. For practical reasons, we were unable to use a “full” grounded theory emergent design in our study. Had we been able to, we might have been able to refine our interview questions and identify a need to pose additional or replacement questions from those used here.

Because all of the participants volunteered to participate in the study, we assume that ours was a well-motivated study group. This selected group of people may already have some pre-existing interest and knowledge about health and physical activity. However, the fact that the possibility of participation was first presented to the management at each workplace raises the risk that the workers felt some pressure to join the research program. If management is positive about health promoting actions, a worker might have felt it difficult to decline participation.

Of the 80 people in the Inphact Treadmill Study, we invited participants from the intervention group to be interviewed as soon as possible after the conclusion of the intervention, and 20 volunteered to take part. Thus, we used a convenience sampling for our interviews and cannot say if we received the most positive participants or those who wanted to complain about the intervention. However, from the analysis, we believe we obtained a good variation with recurrent items regarding their experiences of the treadmill workstations. We consider that additional interviews would not have added any further significant information related to our study objective.

A strength of the study is the triangulation used to increase the reliability and credibility of the results. Two students and two researchers (all within the field of physiotherapy) carried out the interviews. An interdisciplinary team of four authors performed the initial and preliminary coding, two of whom had not participated in the interviews. Each of them coded the interviews individually for later comparison of the codes together, and then searched for emerging patterns in the coding until reaching agreement. The final coding, carried out by a senior in the coding team, was a development of–but still in accordance with–the initial coding, and all co-authors who had participated in the triangulation process recognized and agreed to the interpretations.

## Conclusions

We have observed a core category, the “Ability to benefit,” and four categories delineated as ideal types: the *Convinced*, the *Competitive*, the *Responsible*, and the *Vacillating*. These ideal types corresponded to different strategies and paths, resulting in different abilities to benefit from the intervention. The prompts for behavioral change that work for one ideal type path and strategy may not work for another one. Our results might be transferred to better understand motivation and behavior in similar situations and contexts and create new hypotheses of how interventions with treadmill workstations preferably should be performed. Future interventions with treadmill workstations should, apart from incorporating strategies at the environmental level, also consider using different strategies for different ideal types to obtain the best adherence to these interventions, implying the complexity of such interventions.

## Supporting information

S1 Table The semi-structured interview guide. (DOCX)
