# Supplementary material for: The ability to benefit from an intervention to encourage use of treadmill workstations: Experiences of office workers with overweight or obesity
Source: PLoS One. 2020 Jan 28;15(1):e0228194. doi: 10.1371/journal.pone.0228194 (PMC6986715; doi:10.1371/journal.pone.0228194)
Supplement: S1 Table — (DOCX) [file pone.0228194.s001.docx]

**S1 Table.** **The semi-structured interview guide.**

| **Topic** | **Questions** |
| --- | --- |
| **1. What were your expectations before the start of the study?** | What were your expectations? |
| **2. How have you experienced walking on/using the treadmill during the year?** | - If good: what has been good? - If bad: what has been bad? - How do you rate the physical effort of walking on the treadmill? - How much would you say that you used the treadmill, on average, each work day? - How do you feel your work tasks went while using the treadmill? - How has the treadmill affected your work performance? - What do you think would be required to get office workers to use the treadmill without being part of this research study? |
| **3. What influenced you to use the treadmill?** | - If short answers are given, develop them; certain points in time, situations, work tasks? |
| **4. What stopped you/hindered you from using the treadmill?** | - If short answers are given, develop them; tell me more, certain events, points in time, work tasks? - What would have made you use the treadmill more? |
| **5. Has the treadmill had any effect on your health?** | - In what ways do you feel the treadmill has affected your activity levels outside of work? - How did you feel about the “boosting emails”? Did they affect your usage of the treadmill? - How did you feel about the health consultation that you received at the beginning of the study? Did it affect your usage of the treadmill? - How did you feel about the food diary? |
| **6. Is there anything else that you would like to add or change?** |  |
